# Assessing the COVID‐19 Impact on Air Quality: A Machine Learning Approach

**DOI:** 10.1029/2020GL091202

**Published:** 2021-02-16

**Authors:** Yves Rybarczyk, Rasa Zalakeviciute

**Affiliations:** ^1^ The Department of Data and Information Sciences Dalarna University Falun Sweden; ^2^ SI2 Lab Universidad de Las Americas Quito Ecuador; ^3^ Grupo de Biodiversidad Medio Ambiente y Salud (BIOMAS) Universidad de Las Americas Quito Ecuador

**Keywords:** air pollution, COVID‐19, quarantine measures, urban air quality

## Abstract

The worldwide research initiatives on Corona Virus disease 2019 lockdown effect on air quality agree on pollution reduction, but the most reliable method to pollution reduction quantification is still in debate. In this paper, machine learning models based on a Gradient Boosting Machine algorithm are built to assess the outbreak impact on air quality in Quito, Ecuador. First, the precision of the prediction was evaluated by cross‐validation on the four years prelockdown, showing a high accuracy to estimate the real pollution levels. Then, the changes in pollution are quantified. During the full lockdown, air pollution decreased by −53 ± 2%, −45 ± 11%, −30 ± 13%, and −15 ± 9% for NO_2_, SO_2_, CO, and PM_2.5_, respectively. The traffic‐busy districts were the most impacted areas of the city. After the transition to the partial relaxation, the concentrations have nearly returned to the levels as before the pandemic. The quantification of pollution drop is supported by an assessment of the prediction confidence.

## Key Points

- A data driven modeling is applied to quantify the reduction of air pollution during the Corona Virus disease 2019 outbreak in Quito, Ecuador
- The accuracy of the models is high (mean PCC = 0.78), especially for predicting NO_2_ (mean PCC = 0.87) and CO (mean PCC = 0.86)
- The average drop of pollution during the lockdown is: −53% for NO_2_, −45% for SO_2_, −30% for CO, and −15% for PM_2.5_
- The industrial areas are less impacted by the quarantine than the traffic and residential districts
- The concentration of pollution tends to return to usual levels, as soon as the relaxed restriction is implemented

## 1 Introduction

Throughout 2020, human population of the world faced a challenging new pandemic of Corona Virus disease 2019 (COVID‐19), propagated by a severe acute respiratory syndrome corona virus 2 (SARS‐CoV‐2). Originating in China at the end of 2019, this complex‐symptom disease exponentially spread to almost every part of the world (Worldometer, 2020). In order to slow down social interaction, the principal mode of infection, a range of regulatory measures has been implemented across the countries. Anthropogenic activities were limited or even prohibited, which in turn, positively affected the environmental quality, especially, due to the reduction in emissions by transportation and industries (Shi & Brasseur, 2020; Y. Wang et al., 2020). This was a unique global experiment, as the urban, regional and global air quality conditions have been persistently worsening due to a rapid increase in polluting sources by human population (Limb, 2016; UN., 2015; WHO., 2016). Most common atmospheric contaminants, such as particulate matter (PM, especially with aerodynamic diameters ≤2.5 µm, PM_2.5_), nitrogen oxides (NO and NO_2_), sulfur dioxide (SO_2_), and carbon monoxide (CO), compromise respiratory and cardiovascular systems (EPA, 2018; Lelieveld et al., 2015; Pope et al., 2006). Moreover, a number of studies show that population exposed to a long‐term elevated levels of air pollution have higher mortality rates due to COVID‐19 (Coker et al., 2020; Geographic, 2020; Ogen, 2020; Wu et al., 2020).

Undoubtedly, nearly globally implemented social distancing and isolation measures have resulted in a significant reduction of air pollution (Bauwens et al., 2020; Ding et al., 2020; European Space Agency, 2020; NASA, 2020; Navinya et al., 2020; Nikkei Asian Review, 2020; Querol et al., 2007; Shi & Brasseur, 2020; P. Wang et al., 2020). These statistics of air quality improvements are commonly reported by comparing the restricted human activity periods with the “normal conditions,” months or years before the sanitary emergency with no way to estimate the reliability of these results.

Ecuador is among the countries that implemented extreme quarantine measures. The country had a first reported case of COVID‐19 on February 29, 2020, very quickly escalating in numbers, and in two weeks reporting the first deaths (Worldometer, 2020). In order to prevent health care system collapse, extremely strict national preventative rules were communicated on March 15, active from March 17, 2020. The full lockdown only permitted circulation for absolute necessities (e.g., medicine and food) once per week, based on the license plate number, 7:00–14:00 during the workdays (Zalakeviciute et al., 2020a). Gradually, however, more businesses could function every day anytime. While the infection numbers kept growing, the relaxation to these rules was implemented starting June 2, 2020. Partial relaxation allowed private vehicle circulation every second day, excluding Sundays. This rule permitted all bus functioning (public and private lines) at a limited capacity for passengers. A further increase in permits to some offices were issued (shopping malls, governmental offices, private businesses, etc.).

High elevation capital Quito is characterized by the rainy and dry seasons, with a strong influence of El Niño phenomenon, affecting air pollution conditions, usually worsening during the rainier months (Zalakeviciute et al., 2018). In addition, due to technological advances and increasing strictness of traffic regulations, air quality tends to improve over time, regardless of the motorization trend (European Environment Agency, 2018, 2017; Zalakeviciute et al., 2018). As a result, the evaluation of urban pollution reduction during the sanitary quarantine based on pollution levels from previous years or months could introduce some uncertainties. To get a more accurate assessment of the effect of an event on air quality, a variable normalization technique can be applied (Grange et al., 2018; Grange & Carslaw, 2019). A meteorology‐normalized approach was used to analyze the effect of the COVID‐19 lockdown on NO_2_ concentrations in Spain (Petetin et al., 2020) and in 20 North American cities (Goldberg et al., 2020).

This paper proposes an alternative approach, which consists of training a machine learning (ML) algorithm that will learn the effects of the climatic conditions and time on the atmospheric pollution. ML approach is preferred over chemical transport models, as the latter show a reduced performance in the complex terrain regions (Pani et al., 2020; Žabkar et al., 2015) and require an updated emissions inventory, which Quito lacks. Unlike the previous studies, NO_2_, SO_2_, CO, and PM_2.5_ are analyzed, in the Ecuadorian capital. Since the lockdown affected essentially the human mobility, we investigate the differences in the reduction of pollution in different districts with varied sources of contamination (e.g., traffic vs. industry). One model for each city area and contaminant is built, in order to estimate the concentration of pollutants from meteorological and temporal features without the lockdown. The quantification of the concentration changes due to the reduced human activity, is obtained by comparing the value provided by the model to the real measurements.

## 2 Materials and Methods

### 2.1 Study Site

Ecuador, even though one of the smallest countries in South America, occupies a variety of biomes, including Pacific Coast region, Galapagos Islands Archipelago, Andes Cordillera and the Amazonian Rainforest. The capital Quito is set on a slender plateau, stretching on the side of the Pichincha volcano (elev. 4,800 meters above sea level (m.a.s.l.)), forming a part of the Andes Cordillera. Quito is the most elevated constitutional capital of the world (elev. 2,815 m. a.s.l.) (EMASEO., 2011). The quickly spreading and densifying metro area houses a population of 2.2 million people (INEC., 2011). As most of the cities in the developing countries, Quito reports a long‐term air quality problem (Zalakeviciute et al., 2018).

The Secretariat of Environment of Metropolitan District of Quito manages an air quality network recording atmospheric pollutants and meteorological parameters, since 2004. Six sites with more complete data have been chosen for this study: urban traffic‐busy site Belisario (elev. 2,835 m. a.s.l, coord. 78°29′24″W, 0°10′48″S), urban/industrial sites Camal (elev. 2,840 m. a.s.l, coord. 78°30′36″W, 0°15′00″S), and Carapungo (elev. 2,660 m. a.s.l, coord. 78°26′50″W, 0°5′54″S), suburban/residential traffic site Cotocollao (elev. 2,739 m. a.s.l, coord. 78°29′50″W, 0°6′28″S), suburban/agricultural site Guamani (elev. 3,066 m. a.s.l., coord. 78°33′5″W, 0°19′51″S), and suburban/industrial site Chillos (elev. 2,453 m. a.s.l, coord. 78°27′36″W, 0°18′00″S). More details on the study sites can be found in a previous study evaluating pollution reduction in Ecuador during the first month of COVID‐19 quarantine (Zalakeviciute et al., 2020a).

### 2.2 Quito Air Quality Monitoring

The atmospheric pollution monitoring equipment were set up, following the procedures established by the Environmental Protection Agency of the United States (USEPA). CO concentrations were measured using ThermoFisher Scientific 48i (EPA No. RFCA‐0981‐054). SO_2_ was measured using ThermoFisher Scientific 43i high level SO_2_ analyzers (EPA No. EQSA‐0486‐060). NO_2_ was measured using ThermoFisher Scientific 42i NOx analyzers. PM_2.5_ was measured using Thermo Scientific FH62C14‐DHS continuous ambient particulate monitors 5014i (EPA No. EQPM‐0609‐183).

To register meteorological parameters, complete automatic weather stations were collocated with the atmospheric pollution equipment at study sites. MetOne instruments were used to record wind speed and direction. Thies Clima were employed to measure relative humidity, temperature, and precipitation. Kipp & Zonen radiometers were used to measure solar radiation. Vaisala equipment were used to measure atmospheric pressure.

### 2.3 Machine Learning Modeling

ML models are trained to predict the pollutant concentrations from meteorological and time variables. Seven meteorological features are selected: relative humidity, precipitation, temperature, solar radiation, pressure, wind speed, and wind direction. The time attributes are: Julian day, week day, hour, and trend (date index). These temporal variables account for cyclical emission patterns, which are robust drivers of the concentration of several air pollutants (Henneman et al., 2015). Julian day, weekday, and hours inform respectively on the seasonal (wet vs. dry season), weekly (workday vs. weekend), and hourly (rush hours) occurrences of pollution peaks. Trend reflects the pollution reduction in Quito due to the implementation of fuel and traffic regulations (Zalakeviciute et al., 2018). The features to predict are the concentrations of NO_2_, SO_2_, CO, and PM_2.5_ in the six different districts. We work on hourly data from January 1, 2016 to June 30, 2020. The data set is split in three parts. Data from January 1, 2016 to January 15, 2020 are used to train the model, based on a five‐fold cross‐validation. Then, two months before the full lockdown (January 16, to March 15, 2020) are used to test the prediction accuracy. Even though the COVID‐19 quarantine measures were officially applied starting March 17, the city was showing the lockdown signs starting the March 15, the day of national regulation communication. The Root Mean Squared Error (RMSE) and the Pearson Coefficient of Correlation (PCC) are the metrics chosen to assess the model performance. To validate a model, the RMSE must be minimal, and the PCC the closest to 1. Finally, the remaining data consist of the values during the full ( March 16 to June 1, 2020) and relaxed (June 2 to June 30, 2020) lockdown. The modification of the concentration of contaminants is quantified by subtracting the model predicted values from the actual values recorded during the quarantine.

Due to the complex nonlinear relationships between meteorology and air quality, we selected a nonparametric ML method. A Gradient Boosting Machine (GBM) was chosen, because it is a state‐of‐the‐art decision tree‐based ensemble method (Friedman, 2001). GBM builds an ensemble of shallow and weak successive trees with each tree learning and improving the previous one. As any boosting method, this algorithm adds sequentially new models to the ensemble. At each iteration, a new weak base‐learner model is trained from the error of the entire learned ensemble. A model is considered weak if its error rate is just slightly better than random guessing. In boosting, a weak model is built at each iterative sequence, in order to improve the remaining errors. The generalization of the algorithm is a stagewise additive model of *n* individual regression trees Equation 1.

(1) $$f\left(x\right)=\sum_{n=1}^{N}f^{n}\left(x\right)$$

GBM offers several advantages over the other machine learning algorithms. First, the data set does not need any specific preprocessing. Second, the algorithm handles missing values, and does not require data imputation. Third, since it is based on a decision tree method, feature selection is automatically embedded in the algorithm. Finally, it is possible to identify the importance of each variable in the resulting model.

## 3 Data

Four years and six months of atmospheric pollution and meteorological data were processed on the period prior and during the COVID‐19 quarantine. For each contaminant, the concentration change is computed from the mean difference between ML‐based business‐as‐usual and actual value measured during the outbreak. The H_2_O‐R package was used to implement the GBM algorithm in RStudio. We tuned the parameters of the library so the training (i.e., incrementation of the number of trees) stops after no improvement on the cross‐validated error is provided by 10 consecutive trees. The accuracy of the GBM models is compared to a benchmark, which consists of calculating the average concentration of each pollutant at each site from January 16 to June 30, during 2016–2019. This period is chosen, because the ML models are built on the same years. For illustrative purposes, hourly NO_2_, SO_2_, CO, and PM_2.5_ concentrations were compiled as 24‐h averages starting January 2020. Graphics were done using Microsoft Excel (MS Office) and Igor Pro (Wavemetrics) softwares. The data are available at https://data.mendeley.com/datasets/trs5j932s8/1 (Zalakeviciute, et al., 2020b).

## 4 Results and Discussion

### 4.1 Models and Performance

Twenty‐four models (six sites × four contaminants) were built to predict the level of pollution without lockdown (business‐as‐usual). Table 1 indicates the number of trees used to implement this decision tree‐based ensemble method. More than 6,000 trees were necessary to build the models. The smallest and highest number of trees were in Belisario (mean = 7,283) and Cotocollao (mean = 11,943), respectively.

**Table 1:**
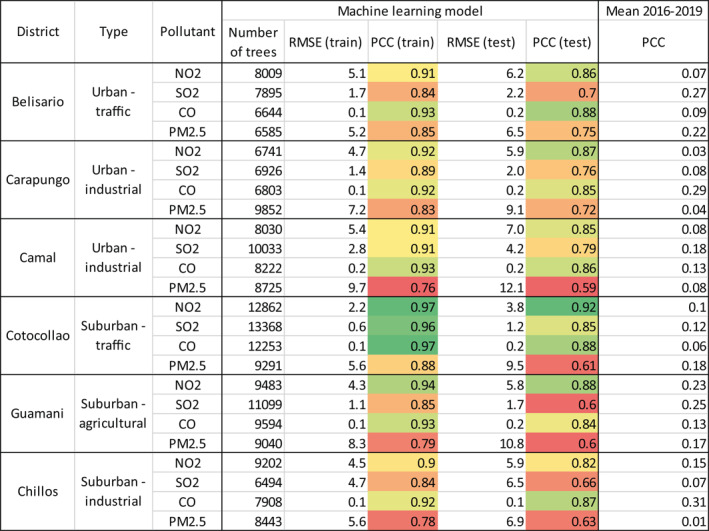
Comparison of the predictive accuracy for the machine learning‐based models (eight column) versus the four previous year average (ninth column).

The metrics used to evaluate the performance of the models (mean RMSE and mean PCC) show a high prediction accuracy. Also, the fact that the performance is systematically lower on the testing set (RMSE = 4.5; PCC = 0.78) than the training set (RMSE = 3.4; PCC = 0.89) suggests no overfitting. The best prediction on the testing set is obtained for NO_2_ (RMSE = 5.8; PCC = 0.87) and CO (RMSE = 0.2; PCC = 0.86). The model accuracy for SO_2_ is good (RMSE = 3; PCC = 0.73) and fair for PM_2.5_ (RMSE = 9.1; PCC = 0.65). For these last two pollutants, there are more accuracy differences from one district to another (stddev PCC SO_2_ = 0.08; stddev PCC PM_2.5_ = 0.06) than for the best predicted gases (stddev PCC NO_2_ = 0.03; stddev PCC CO = 0.01). These results show that the reliability of the proposed ML‐based models to predict the concentration of pollutants is higher for NO_2_ and CO than SO_2_ and PM_2.5_. The prediction of the first three contaminants is better than PM_2.5_. Those are contaminants strictly related to the fossil fuel combustion, and in an urban area they are directly resulting from human activity. On the contrary, PM_2.5_ while also mostly emitted by mobile sources, it may come from other sources (Karagulian et al., 2015), settle on the surfaces and disperse again in the atmosphere due to wind ventilation.

On the other hand, a benchmark based on the averages of four previous years shows a very low accuracy. Its correlation with the values observed from mid‐January 2020 to mid‐March 2020 does not exceed 0.3 (PCC NO_2_ = 0.11; PCC CO = 0.17; PCC SO_2_ = 0.16; PCC PM_2.5_ = 0.12). This result suggests that applying a simple average of the concentration measured in 2016–2019, for the lockdown period, is not reliable to quantify the impact on air quality. This outcome highlights the benefit of building a model based on an ML approach, particularly because it considers actual meteorological conditions and temporal features.

It is to note that the model accuracy is also dependent on the characteristics of the metropolitan areas. The best prediction is obtained for the districts in which the source of pollution is traffic‐based (PCC for Belisario = 0.8; men PCC for Cotocollao = 0.82). The performance is slightly worse for the industrial (PCC for Carapungo = 0.8; PCC for Camal = 0.77; PCC for Chillos = 0.75) and agricultural areas (PCC for Guamin = 0.73). Whether the district is urban (PCC = 0.79) or suburban (PCC = 0.77) does not influence significantly the quality of the prediction. This is because both areas are circulated by the same poor‐quality fuel‐powered buses and private vehicles. These results can be explained by the fact that the main source of pollution in Quito is traffic‐based, especially peaking during rush hours (Zalakeviciute et al., 2018). These precise patterns can be learned by our data‐driven models. Also, a possible distinction between inner‐city and suburb is not verified, because the COVID‐19 quarantine regulations are applied extremely strictly in all the metropolitan area.

### 4.2 Variable Importance

**Figure 1:**
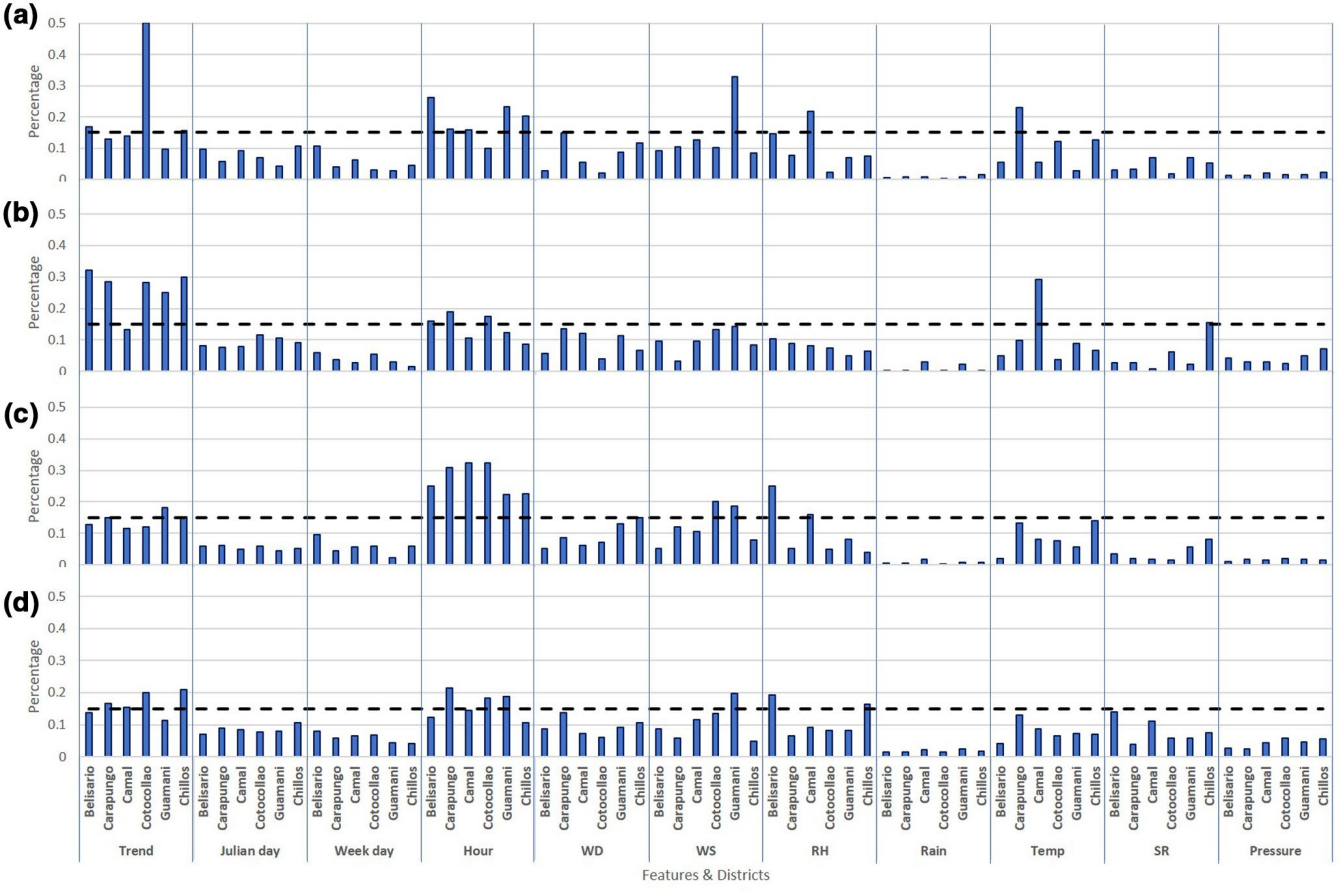
Variable importance for the modeling of NO_2_ (panel (a)), SO_2_ (panel (b)), CO (panel (c)), and PM_2.5_ (panel (d)) for the six districts. The broken line is an arbitrary threshold (15% of importance) used to highlight the most relevant features for the prediction of each contaminant. WD, WS, RH, Temp and SR stand for wind direction, wind speed, relative humidity, temperature and solar radiation, respectively.

GBM algorithm allows us to understand how the features have been handled by the resulting models. The importance of the 11 independent variables for the six districts and four pollutants is represented in Figure 1. For a determined contaminant and site, this importance is expressed in percentage of each feature. If 15% is used as minimal threshold, the five more relevant factors to build the models are: trend, hour, wind speed (WS), relative humidity (RH), and temperature (Temp). The other six features do not exceed this cut‐off whatever the district and the pollutant. The two temporal features, trend and hour, are always among the most important variables for the modeling of any contaminant. For the trend feature, it can be explained by the implementation of the successive regulations, which have progressively improved air quality in Quito over the last decade (Zalakeviciute et al., 2018). As previously mentioned, the daily pollution peaks occur during the morning and evening rush hours systematically making hour a reliable feature to predict the concentration of anthropogenic pollutants.

We note that the three most relevant meteorological features (WS, RH, and Temp) have, on average, a lower percentage of importance than the two temporal factors (Trend and Hour). WS is mostly used for the prediction of PM_2.5_ and CO. The transport and re‐suspension effect of wind on the traffic‐related contamination can explain the importance of this factor in the models. Similarly, RH seems to influence mainly the concentration of these two pollutants. A previous study by the authors shows that wet conditions increase traffic‐related pollution, because humidity reduces engine efficiency, especially in high‐elevation cities (Zalakeviciute et al., 2018). Among the three relevant meteorological factors, temperature is the least important. Nevertheless, it is significantly used for the modeling of NO_2_ in Carapungo and SO_2_ in Camal, which are both urban‐industrial areas. Temperature, which is positively correlated with WS and negatively correlated with RH, is known for causing thermal turbulences that contribute to dilute gas emissions.

In terms of districts, some unique percentages of importance emerge. For Cotocollao, trend constitutes half the features for predicting the NO_2_ concentration. It means that the consecutive fuel and traffic regulations have largely impacted this area. On the contrary, the level of NO_2_ and PM_2.5_ in the suburban agricultural site of Guamani are more influenced by wind velocity, suggesting transport factor. Camal, as an urban‐industrial area, is the only district which exhibits concentration levels of SO_2_ more affected by temperature than the trend feature, also suggesting increased temperature effect on pollution transport. Considering the dense road network in Belisario, it was expected to get RH with high percentage of importance for modeling PM_2.5_ and CO in this urban area. For CO, directly linked to combustion sources, hour is systematically the most important feature independently of the district.

### 4.3 Effect of the Mobility Restrictions

**Figure 2:**
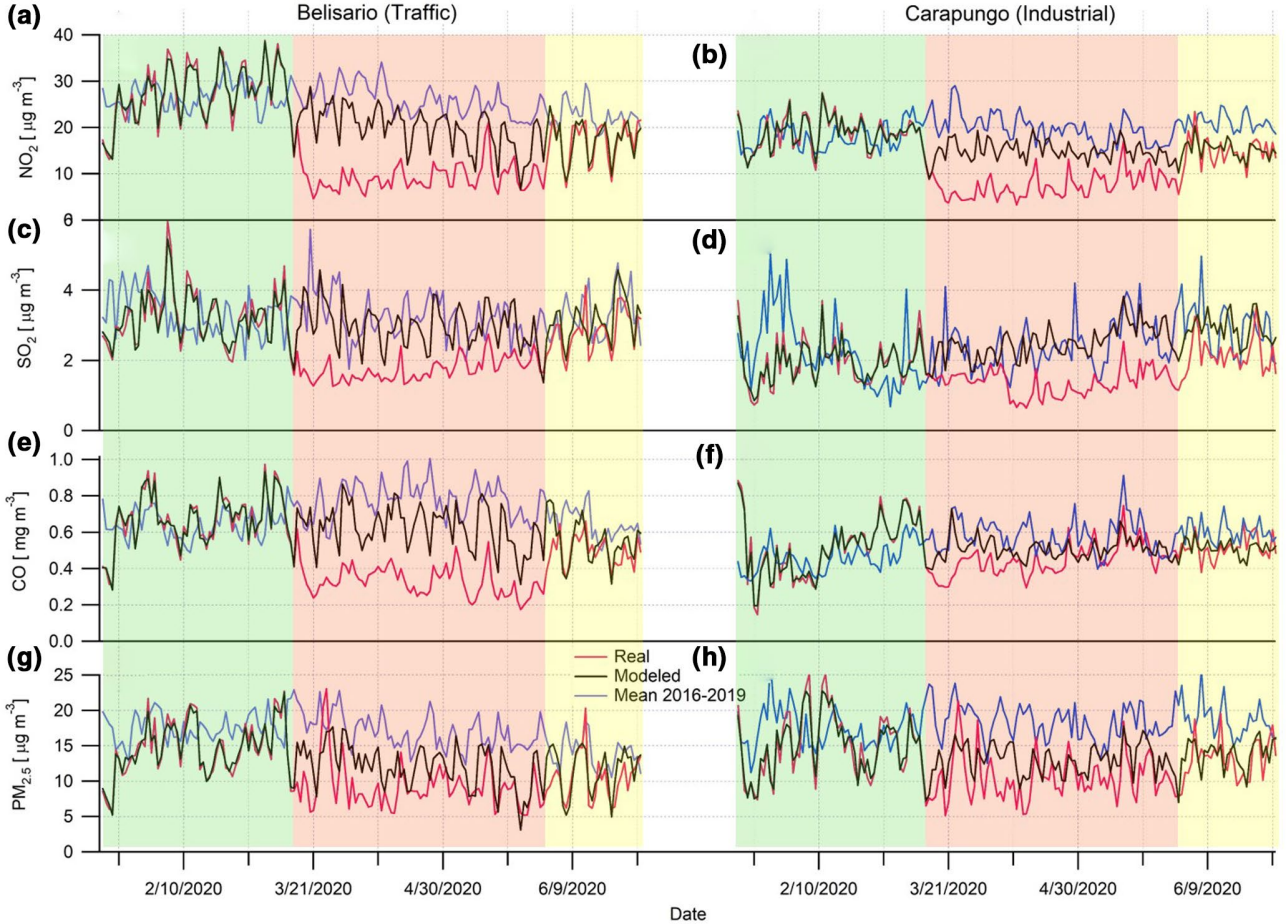
Real and estimated (ML modeled and four previous year mean) concentration of pollutants in traffic (Belisario) and industrial (Carapungo) areas during the prepandemic (green‐shaded area), full‐lockdown (red‐shaded area), and partial relaxation (yellow‐shaded area). For Belisario, panels (a), (c), (e) and (g) represent the concentrations of NO_2_, SO_2_, CO and PM_2.5_, respectively. For Carapungo, NO_2_, SO_2_, CO and PM_2.5_ are plotted in panels (b), (d), (f), and (h), respectively. ML: Machine Learning.

Figure 2 represents the concentration of the four pollutants before (green‐shaded area), during (red‐shaded area) and after the full lockdown (yellow‐shaded area), for two types of districts (traffic vs. industrial). A clear drop of concentrations is observed from the beginning of the lockdown (March 16, 2020), especially for NO_2_, SO_2_, and CO (Figure 2, panels a–f). The measurements are a bit noisier for PM_2.5_, and the reduction is not so obvious in both, traffic (Belisario) and industrial (Carapungo) areas (Figure 2, panels g–h). For instance, high PM_2.5_ concentrations can be noticed around 20 March. This peak seems to be caused by windy conditions, possibly re‐suspending fine particulate matter into the atmosphere (Csavina et al., 2014). The contrasted and limited effect of the lockdown on the concentration of PM_2.5_ is also observed in other cities around the world due to regional pollution events or an increase in private vehicles' use (Dhaka et al., 2020; Faridi et al., 2020). Another explanation is the fact that PM are not only emitted by vehicles, but also factories which continued to work almost normally during the quarantine. The relatively low reduction of PM_2.5_ in the industrial districts of Quito (−20% for Camal, −19% for Carapungo, and +3% for Chillos) supports this hypothesis (Table 2). Once the full lockdown is over (June 1, 2020), the concentrations return progressively to close to the same levels as before the pandemic.

**Table 2:**
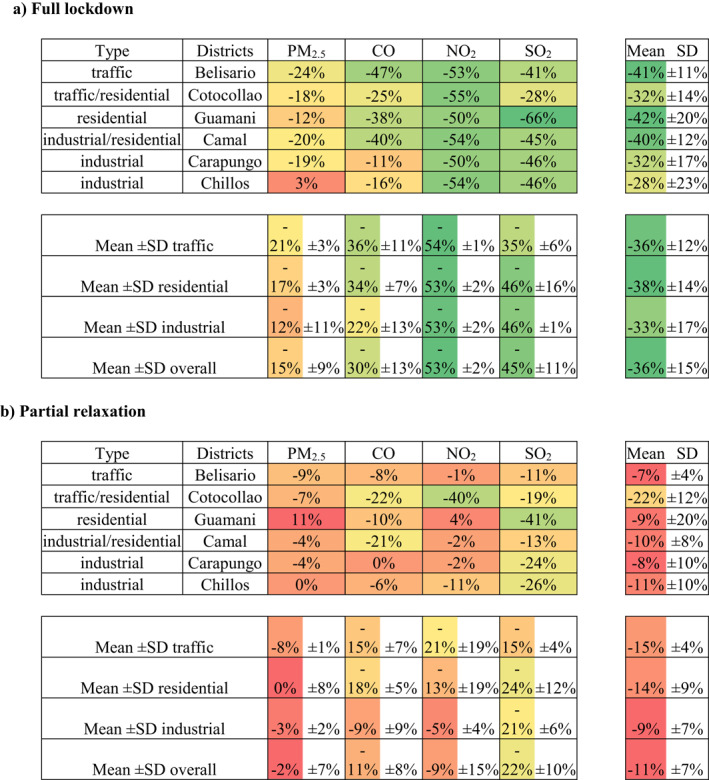
Effect of the two levels of mobility restriction on the concentration of pollutants: (a) full‐lockdown, and (b) partial relaxation.

*Note:* Color scale from red to green highlights the percentage of pollution reduction. The greener is the cell, the higher is the pollution drop. SD stands for standard deviation.

Moreover, Figure 2 shows the accuracy of the estimation of the pollution levels as if no lockdown was implemented. Before March 16, the overlap between predictive data, from the ML models, and actual data is almost perfect. It suggests that we can reasonably trust these predictive values as very close to the ones that would have been recorded if the human activities were usual. On the contrary, a reference based on the average of four previous years does not correlate at all with the real data. This visualization confirms that the proposed GBM‐based models can provide an accurate benchmark to quantify the pollution drop during the lockdown. The estimation of pollution reduction provided by the ML approach is by far more reliable than a simple statistical averaging of the value recorded during the past years. The robustness of the GBM models is verified by the fact that actual and predictive data re‐overlap progressively after the implementation of the partial relaxation measures.

Summarizing, during the full lockdown, NO_2_ gets the highest drop, reducing by half its concentrations (overall mean: −53% ± 2%), independently of the district (Table 2a). SO_2_ is also strongly reduced over the whole city (overall mean: −45% ± 11%), followed by CO (−30% ± 13%). CO decreases more in the traffic and residential areas (around 35%) than in the industrial areas (around 20%), which can be explained by the fact that CO is directly emitted from vehicles. As previously mentioned, the effect of the lockdown on PM_2.5_ is less important, but more significant for traffic‐busy than industrial districts. For instance, the level of PM_2.5_ kept constant in Chillos, but more reduced in traffic‐busy areas, because this anthropogenic pollutant is mostly emitted by diesel vehicles. In the case of Chillos, it comes from thermoelectric power plant and factories, which maintained their activity during the pandemic.

When the restrictions on human mobility subsided slightly, the atmospheric pollution returned to almost normal levels (overall mean: −11% ± 7%, during the partial relaxation, against −36% ± 15%, during the full lockdown), especially for PM_2.5_, CO, and NO_2_ (Table 2b). Even if the reduction was not comparable with the lockdown period, SO_2_ levels stayed lower than usual. There are also certain inconsistencies from one site to another (e.g., −40% NO_2_ in Cotocollao, against +4% in Guamani) and from one pollutant to another (e.g., +11% PM_2.5_, against −41% SO_2_ in Guamani). The consequences on air quality are a bit noisier during the partial relaxation due to a more stochastic human activity.

## 5 Conclusions

This study aimed to estimate the impact of COVID‐19 on NO_2_, CO, PM_2.5_, and SO_2_ in Quito, Ecuador. A GBM algorithm was used to build 24 models to predict the air quality as if COVID‐19 quarantine never existed. Unlike other weather normalization studies focusing on a single pollutant, we addressed the prediction of more pollutants and investigated the importance of more variables. The temporal features were among the most important variables for the modeling of any contaminant, while meteorological parameters are pollutant‐ and site‐dependent.

The accuracy of the models was evaluated through a cross‐validation on several years before the lockdown, starting January 2016. The evaluation metrics show a high predictive performance for NO_2_ and CO, a good one for SO_2_ and fair for PM_2.5_, varying for different districts in the city. The best model accuracy is obtained for the traffic‐busy areas. This study suggests that previous years' average concentrations are not as reliable in estimating the effect of human activities on air quality and highlights the benefit of building a model based on an ML approach, considering actual meteorological conditions and temporal features.

Finally, the lockdown effect on air pollutants was analyzed for traffic and industrial districts. A clear reduction is observed for NO_2_ (−53% ± 2%), SO_2_ (−45% ± 11%), and CO (−30% ± 13%) concentrations during the full lockdown. The reduction in PM_2.5_ concentrations display some variability depending on the type of monitoring site (traffic site: −21% ± 3%, industrial site: −12% ± 11%). Weather events, like increased wind speed, help explain the reduced effect of mobility regulations on PM_2.5_. Unavoidably, implementing partial relaxation measures, the pollution concentrations progressively return to close to the same levels as before the pandemic.

Months into the pandemic, the world is overwhelmed with the implications to the economic development, public health, mobility, and environmental politics. However, this short break in a production of anthropogenic emissions showed a different reality with a global reduction of pollution. This unexpected scenario was an opportunity to assess the weight of the human activity (especially its mobility) on the atmospheric environment (Turner et al., 2020). The sanitary emergency highlighted the importance of strict traffic policies to improve urban air quality and reduce emission of greenhouse gases. In that sense, future work must be carried out on the impact of human mobility on the atmospheric pollution in the rapidly growing developing countries and an efficient transition to cleaner transportation. Although this study focuses on the COVID‐19 pandemic, the reliability of our predictions supports the idea that an ML‐based modeling is a robust method to quantify the impact of any event on air pollution.

## Data Availability

There are no real or perceived financial conflicts of interests for any author.The data supporting the conclusions can be obtained at Mendeley Data, V1, doi: 10.17632/trs5j932s8.1. (Zalakeviciute, et al., 2020b), “Air Quality in Quito during COVID‐19 outbreak” at https://data.mendeley.com/datasets/trs5j932s8/1
